# Prevalence of heart failure pharmacotherapy utilisation, frailty and adverse drug events among hospitalised adults older than 75 years: a multicentre cross‐sectional study

**DOI:** 10.1111/imj.16612

**Published:** 2024-12-19

**Authors:** Mai H. Duong, Danijela Gnjidic, Andrew J. McLachlan, Kevin Winardi, Alexandra A. Bennett, Fiona Blyth, David Le Couteur, Sarah N. Hilmer

**Affiliations:** ^1^ Laboratory of Ageing and Pharmacology Kolling Institute, Northern Sydney Local Health District Sydney New South Wales Australia; ^2^ Faculty of Medicine and Health The University of Sydney Sydney New South Wales Australia; ^3^ NSW Therapeutic Advisory Group Sydney New South Wales Australia

**Keywords:** congestive heart failure, polypharmacy, observational study, guideline adherence, drug utilisation, data visualisation

## Abstract

**Background:**

Optimal heart failure (HF) pharmacotherapy (guideline‐directed medical therapy and diuretics) in older people with frailty is uncertain due to limited evidence.

**Aims:**

To evaluate utilisation of HF pharmacotherapy and prevalence of polypharmacy, adverse drug events (ADEs), falls, delirium, renal impairment and duration of hospitalisation in older inpatients, according to frailty.

**Methods:**

A retrospective cross‐sectional study of the TO HOME cohort of 2000 inpatients ≥75 years admitted for ≥48 h to rehabilitation, geriatric or general medicine from 1 July 2016 to 30 June 2017 across six hospitals in Sydney, Australia. Data were collected from electronic medical records. International Statistical Classification of Diseases and Related Health Problems, Tenth Revision, Australian Modification identified HF diagnosis, ADEs and frailty using hospital frailty risk score. Outcomes included utilisation of HF pharmacotherapy; polypharmacy; ADEs, falls, delirium, renal and impairment; and duration of hospitalisation.

**Results:**

Among 439 (22.0% of TO HOME cohort) patients with undifferentiated HF, 284 (69.5%) had intermediate or high risk of frailty, and 412 (94%) took ≥1 HF pharmacotherapy, with 357 (81.3%) patients on loop diuretics. Patients with high frailty risk frequently continued beta‐blockers (70%) and discontinued renin‐angiotensin system inhibitors (57%). Most patients experienced polypharmacy (*n* = 426, 97.0%). Renal impairment prevalence was 67%–76% across frailty groups. Increasing frailty risk (low, intermediate and high) was associated with increasing prevalence of ADEs (31%, 56% and 84%), falls (12%, 25% and 46%) and delirium (8%, 27% and 49%) and longer hospitalisation.

**Conclusions:**

Frailty, HF‐pharmacotherapy changes in hospital and ADEs were common among older inpatients with HF. The association of adverse outcomes according to frailty needs further investigation. Poor documentation of HF phenotype may be a barrier to medication optimisation in older inpatients.

**Abbreviations**
ADE: adverse drug event
ADR: adverse drug reaction
aHR: adjusted hazard ratio
aOR: adjusted odds ratio
CI: confidence interval
CCI: Charlson comorbidity index
eMR: electronic medical record
GDMT: guideline‐directed medical therapy
GFR: glomerular filtration rate
HF: heart failure
HFpEF: heart failure with preserved ejection fraction (LVEF > 50%)
HFrEF: heart failure with reduced ejection fraction (LVEF < 40%)
HFRS: hospital frailty risk score
ICD‐10‐AM: International Statistical Classification of Diseases and Related Health Problems, Tenth Revision, Australian Modification
LOS: length of stay
MRA: mineralocorticoid receptor antagonist
OR: odds ratio
RASI: renin‐angiotensin system inhibitors
SD: standard deviation
SGLT2‐i: sodium‐glucose cotransporter 2 inhibitors
TO HOME: Towards Optimising Hospitalised Older adults' MEdications

## Introduction

Heart failure (HF) is a complex syndrome increasingly prevalent in the ageing population and associated with high risk of hospitalisation and death.^1^ Half of HF hospitalisations involve people aged ≥75 years.^2^ Frailty, a reversible syndrome involving multisystem deficiencies, affects up to 75% of people living with HF in the community.^3^ Utilisation and optimisation of HF guideline‐directed medical therapies (GMDTs) (i.e. renin‐angiotensin system inhibitors (RASIs), HF‐specific beta‐blockers, mineralocorticoid receptor antagonists (MRAs) and, more recently, sodium‐glucose co‐transporter‐2 inhibitors (SGLT2‐i)) have demonstrated reduced mortality and hospitalisation.^4^ Older people report lower tolerance and higher discontinuation rates of HF GDMTs.^5^, ^6^

Polypharmacy is highly prevalent in patients with HF, especially as their age increases. Ninety‐five per cent take ≥5 medications and 50% take ≥10 medications, increasing the risk of adverse drug reactions (ADRs).^7^ Up to 91% of HF patients report experiencing chronic adverse drug events (ADEs).^8^ Previous studies of hospitalised older adults reported HF in approximately 13% of inpatients,^9^ 40% were affected by frailty and 8.6%–18.4% reported ADRs in hospital.^10^, ^11^, ^12^, ^13^ The prevalence of ADRs in people aged <65 years compared to those ≥65 years differed by 30%–60%.^14^ Hospitalisation of older patients with HF often occurs under the care of multidisciplinary teams, which may or may not include cardiology specialists.

The management of HF and optimisation of medications in frail older people can be complex due to disease progression, changes in cognition, multimorbidity, life expectancy and changing patient priorities. Frail older people are persistently and widely excluded from HF clinical trials, which measure cardiovascular outcomes rather than geriatric outcomes; thus, evidence on efficacy and safety in this population is limited.^15^, ^16^ Research evaluating geriatric outcomes is needed to fill this knowledge gap to support shared decision‐making.

This study aimed to evaluate the utilisation of HF GDMTs and diuretics in hospitalised patients ≥75 years with HF, according to frailty risk. Secondary objectives were to determine the duration of hospitalisation and evaluate the prevalence of polypharmacy and geriatric‐relevant adverse events in patients with HF on HF GDMTs or diuretics at hospital discharge by frailty risk.

## Methods

### Data source

This retrospective cross‐sectional study included 2000 consecutive patients aged ≥75 years admitted for ≥48 h to geriatric, general and rehabilitation medicine between 1 July 2016 a 30 June 2017 across six hospitals in Sydney, Australia. Data were collected from patient electronic medical records (eMRs) in routine care and verified by a research clinician on medications, clinical characteristics and outcomes (e.g. changes in medication utilisation, ADEs, falls, delirium and renal impairment) during admission. Data were obtained from the Towards Optimising Hospitalised Older adults' MEdications (TO HOME) dataset, designed to look at overall medication management during routine care in general medical, geriatric and rehabilitation services, previously published methods and characteristics.^17^ Patients with HF who present with geriatric syndromes or non‐cardiac reasons for admission (e.g. infection, falls), with or without active cardiac diagnosis, are likely to be admitted to general medical and geriatric hospital services for HF or non‐HF admissions. This study did not capture data for patients admitted under cardiology service, more likely to be admitted for initial diagnosis of HF, acute treatment of severe HF requiring critical care or HF without other active diagnoses.

### Study subjects

Patients were identified using coding of diagnoses during hospital admission with the International Statistical Classification of Diseases and Related Health Problems, Tenth Revision, Australian Modification (ICD‐10‐AM) codes. ICD‐10‐AM coding for HF diagnosis and related cardiomyopathy^18^ included undifferentiated HF (U82.2, I50, I50.0, I50.1, I50.9, I09.9, I11.0, I13.0, I13.2, I25.5, I42.0, I42.5–42.9, I43, P29.0, E10.53, E11.53, E13.53, E14.53), heart failure with reduced ejection fraction (HFrEF) (I50.2x, I50.4x) and heart failure with preserved ejection fraction (HFpEF) (I50.3). Demographic characteristics were determined for patients with HF diagnosis compared to those with no HF diagnosis. Previous ADRs were identified from documentation in admission notes and discharge summaries in the eMR.

### Frailty measurement

Frailty risk of each patient was calculated with ICD‐10‐AM codes according to the hospital frailty risk score (HFRS), a numerical score determined by relevant ICD‐10‐AM codes during admission as previously reported.^19^ The HFRS calculates the risk of frailty into three categories: low (<5 points), intermediate (5–15 points) or high (>15 points). All outcomes were reported by frailty risk (HFRS categories) to mitigate reporting bias. This study evaluated the prevalence of medication utilisation and highly prevalent outcomes among geriatric populations with cardiovascular disease, particularly polypharmacy, duration of hospitalisation, ADEs, falls, delirium and renal impairment, according to frailty risk.^20^

### HF pharmacotherapy utilisation

Anatomical Therapeutic Chemical Classification codes for HF‐GDMT^4^ include RASIs (C09), beta‐blockers (C07), MRAs (C03D, C03DA) and loop diuretics (C03, C03CA, C03CA01). The codes for SGLT2‐i (A10BK) were not included in this analysis since they were not recommended in HF guidelines at the time of data collection. RASIs mostly referred to angiotensin converting enzyme inhibitors and angiotensin receptor antagonists as sacubitril/valsartan was not approved in Australia during the entire data collection period. Diuretics include loop diuretics (i.e. furosemide, bumetanide, etacrynic acid) and exclude thiazide diuretics.

The primary outcome measure was HF pharmacotherapy utilisation by individual therapeutic class and combination (i.e. taking at least one GDMT or diuretic). These categorical data are reported as the frequency and proportion of initiation, continuation, uptitration, downtitration, discontinuation and re‐initiation of each class of HF pharmacotherapy at discharge. Analysis did not reveal whether individual doses for each HF pharmacotherapy met the criteria for recommended optimised doses in clinical trials. Polypharmacy (the use of ≥5 regular medications) and hyperpolypharmacy (the use of ≥10 regular medications) at admission and discharge were reported for all HF patients. The frequency of two, three and four HF pharmacotherapy combinations per patient and the prevalence of overall pairwise combinations were reported.

### Clinical outcomes

Secondary outcomes were measured as the frequency and proportion of clinical outcomes, reported as ADEs, falls, delirium and renal impairment for patients taking ≥1 HF pharmacotherapy at discharge. ADEs are defined as any adverse outcome occurring in people taking a medication.^21^ ADEs were identified with ICD‐10‐AM codes using Du *et al*.^22^ published criteria to identify potential ADEs of clinical significance occurring in hospital. Specifically, the combination of ICD‐10‐AM external cause codes (Y40‐59)^23^ and previously published ICD‐10‐AM codes assigned causality ratings (A, B, C and D) estimating the probability that a specific diagnosis was related to an ADR. Falls and delirium were extracted from the free text in the eMR. Renal Impairment (acute or chronic) was defined as glomerular filtration rate <60 mL/min/1.73 m^2^ measured at admission.^24^ Missing data (*n* = 1) for renal impairment identified in one HF patient with intermediate frailty were coded as no renal impairment. Duration of hospitalisation (length of stay (LOS)) was measured as the number of days (mean, standard deviation) between patient initial admission and discharge.

### Statistical analysis

A descriptive analysis was performed on the TO HOME cohort, stratified by frailty risk. Sample size was determined by the TO HOME cohort. A retrospective power analysis was conducted. A clinically significant difference in prevalence of ADRs was considered as 10% in patients aged ≥65 years in a recent systematic review.^25^, ^26^ Therefore, the study had 0.87 power to detect a 10%–18% difference in ADRs from HF pharmacotherapy when comparing people with low, intermediate and high risk of frailty.^11^, ^27^ Descriptive statistics were applied to calculate frequency and proportions of all outcomes. The mean (standard deviation) was reported for continuous variables, and count (%) was reported for categorical variables, binary groups and frailty risk and graphed. Sociodemographic and hospital admission data in patients with and without HF were compared using the Pearson chi‐squared test for categorical variables and the Mann–Whitney *U* test for continuous variables. Differences between patients with HF and no HF diagnosis were deemed statistically significant if the *P*‐value was <0.05. Subgroup analysis reported outcomes by frailty risk. Patterns between frailty groups were not compared statistically. Instead, weighted utilisation for individual therapeutic classes were grouped by frailty risk using radar plots,^28^, ^29^ and heatmaps of HF pharmacotherapy pairwise combinations per person with annotations of frequency and proportions of ADEs were visualised.^30^ Data analysis and visualisation were performed in R/Rstudio version 4.2.2 using ggplot2 (version 3.5.0), ggradar (version 0.2) and ComplexHeatmap (version 2.14.0), along with Microsoft Excel 2014.

## Results

### Demographics

**Figure 1:**
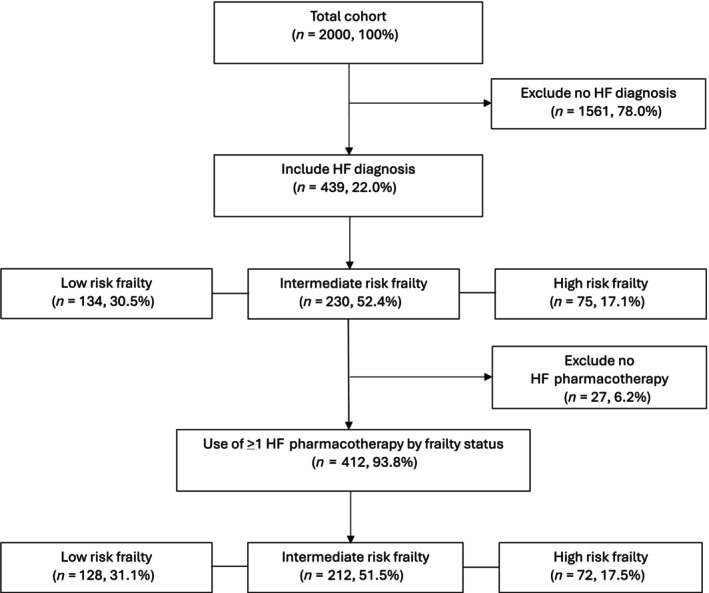
Flow chart of study population selection.

Undifferentiated HF was identified in 439 (22%) patients; no coding reported HFrEF or HFpEF (Fig. 1). Table 1 describes patient characteristics in those with HF compared to no HF diagnosis. The proportion of frailty risk was similar among patients with diagnosis of HF (*n* = 305, 69.5%) and no HF (*n* = 1107, 70.9%). In patients with HF, there was low (*n* = 134, 30.5%), intermediate (*n* = 230, 52.4%) and high (*n* = 75, 17.1%) frailty risk. The mean age was 87 (5.6) years, 59.0% (*n* = 259) were female, 15.3% (*n* = 90) had dementia, 7.1% (*n* = 42) had mild cognitive impairment, 134 (30.5%) and 67.2% (*n* = 295) considered English their preferred language. Compared to patients without HF diagnosis, patients with HF had more comorbidities (Charlson comorbidity index ≥ 3)^31^ (*n* = 134, 30.5% HF, *n* = 201, 12.9% without HF), lower prevalence of documented dementia (*n* = 90, 15.3% HF, *n* = 427, 27.4% without HF) and a higher prevalence of a cardiac or circulatory system‐related primary reason for admission (*n* = 125, 28.5% HF, *n* = 95, 6.1% without HF). Of those with a diagnosis of HF, HF was the primary reason for admission in 97 (22.1%). Prior to admission, 68.8% (*n* = 302) lived at home and 50.1% (*n* = 220) were discharged home.

**Table 1:** Patient characteristics

Characteristics, *n* (%)	Total		Heart failure		No heart failure	
	*n*	%	*n*	%	*n*	%
Sample size	2000	100%	439	22%	1561	78%
Age (years), mean (SD)	86 (5.8)	—	87 (5.6)	—	86 (5.8)	—
75–79	307	15.4%	44	10%	263	17%
80–89	1116	55.8%	245	56%	871	56%
≥90	577	28.9%	150	34%	427	27%
Sex (female)	1181	59.1%	259	59.0%	922	59.1%
Preferred language spoken at home						
English	1381	69%	295	67.2%	1092	70.0%
Language other than English	613	31%	144	32.8%	469	30.0%
Frailty risk (hospital frailty risk score (HFRS))						
Low (HFRS < 5)	588	29.4%	134	30.5%	454	29.1%
Intermediate (HFRS 5–15)	1087	54.4%	230	52.4%	857	54.9%
High (HFRS > 15)	325	16.3%	75	17.1%	250	16.0%
Number of comorbidities (Charlson Comorbidity Index)‡						
Low: score 0–2	1665	83.2%	305	69.4%	1360	87.1%
High: score 3+	335	16.8%	134	30.5%	201	12.9%
Cognition						
Dementia documented‡	517	26%	90	15.3%	427	27.4%
Mild cognitive impairment	197	10%	42	7.1%	155	9.9%
Primary reason for admission						
Cardiac and circulatory system‡	220	11%	125	28.5%	95	6.1%
Heart failure	97	4.9%	97	22.1%	—	—
Low risk (HFRS < 5)	48	2.4%	48	11%	—	—
Intermediate risk (HFRS 5–15)	43	2.2%	43	10%	—	—
High risk (HFRS > 15)	6	<1%	6	1.4%	—	—
Length of hospital stay (days) mean (SD)	13.4 (15.6)	—	13.5 (12.7)	—	13.4 (16.3)	—
History of a previous ADR† prior to admission	831	41.6%	207	47.2%	624	40.0%
Living status prior to admission						
Home	1458	73%	302	68.8%	1156	74.1%
Residential aged care facility	421	25%	108	24.6%	313	20.1%
Other	121	22%	29	6.6%	92	5.9%
Discharge destination						
Home	1050	53%	220	50.1%	830	53.2%
Residential aged care facility	506	25%	123	28.0%	383	24.5%
Assisted living	78	4%	20	4.6%	58	3.7%
Hospice/palliative care	1	<1%	0	0.0%	1	0.1%
Other	365	18	76	17.3%	289	18.5%

† Participant had documented adverse drug reactions.

‡ Difference between HF and no HF was statistically significant (*P* < 0.05).

### HF pharmacotherapy utilisation

The use of at least one HF pharmacotherapy was reported in 412 (94%) patients with a documented diagnosis of HF. At discharge, HF pharmacotherapy was frequently continued (*n* = 289, 65.8%). HF pharmacotherapy withheld or discontinued during hospital admission was commonly not recommended for re‐initiation at discharge, with <1% being recommended for re‐initiation if deemed appropriate by their general practitioner (Fig. S1, Table S1). During hospitalisation, patients with HF were frequently prescribed loop diuretics (*n* = 357, 81.3%, predominantly furosemide (*n* = 355, 80.9%)), MRAs were less frequently prescribed (*n* = 100, 22.8%), and half were prescribed RASIs (*n* = 218, 49.7%) and/or beta‐blockers (*n* = 216, 49.2%) (Table S2). HF‐specific beta‐blockers (e.g. excluding atenolol, labetalol, propranolol, sotalol) were reported for 179 (40.8%) patients. Sotalol, which may exacerbate HF, was prescribed for 11 (2.5%) patients diagnosed with HF but not with HF as the primary reason for admission and was continued (*n* = 8, 1.8%), discontinued (*n* = 2, 0.5%) or downtitrated (*n* = 1, 0.2%) during admission.

**Figure 2:**
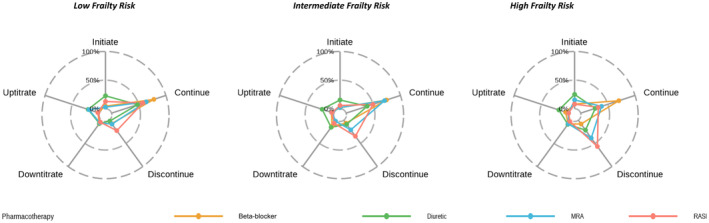
The proportion of heart failure‐pharmacotherapy utilisation in hospitalised patients over 75 years with heart failure, by frailty risk.

Deprescribing of HF pharmacotherapy was frequent (*n* = 185, 42.1%) (Table S1). Of the 97 patients admitted with HF as the primary reason for admission, 22 (22.7%) discontinued and 19 (19.6%) downtitrated HF pharmacotherapy. Discontinuation was most prevalent for RASIs (*n* = 75, 17.1%). Loop diuretics were most frequently adjusted during hospitalisation, as doses were initiated (*n* = 68, 15.5%), uptitrated (*n* = 74, 16.9%) and downtitrated (*n* = 41, 9.3%). Continuation of beta‐blockers (*n* = 160, 36.4%) and diuretics (*n* = 142, 32.3%) were most prevalent across frailty subgroups (Fig. 2). High‐frailty‐ and risk patients frequently continued beta‐blockers (*n* = 26, 70.3%) discontinued RASIs (*n* = 21, 56.8%), and MRAs were similarly continued (*n* = 5, 38.5%) and discontinued (*n* = 5, 38.5%).

### Polypharmacy

**Figure 3:**
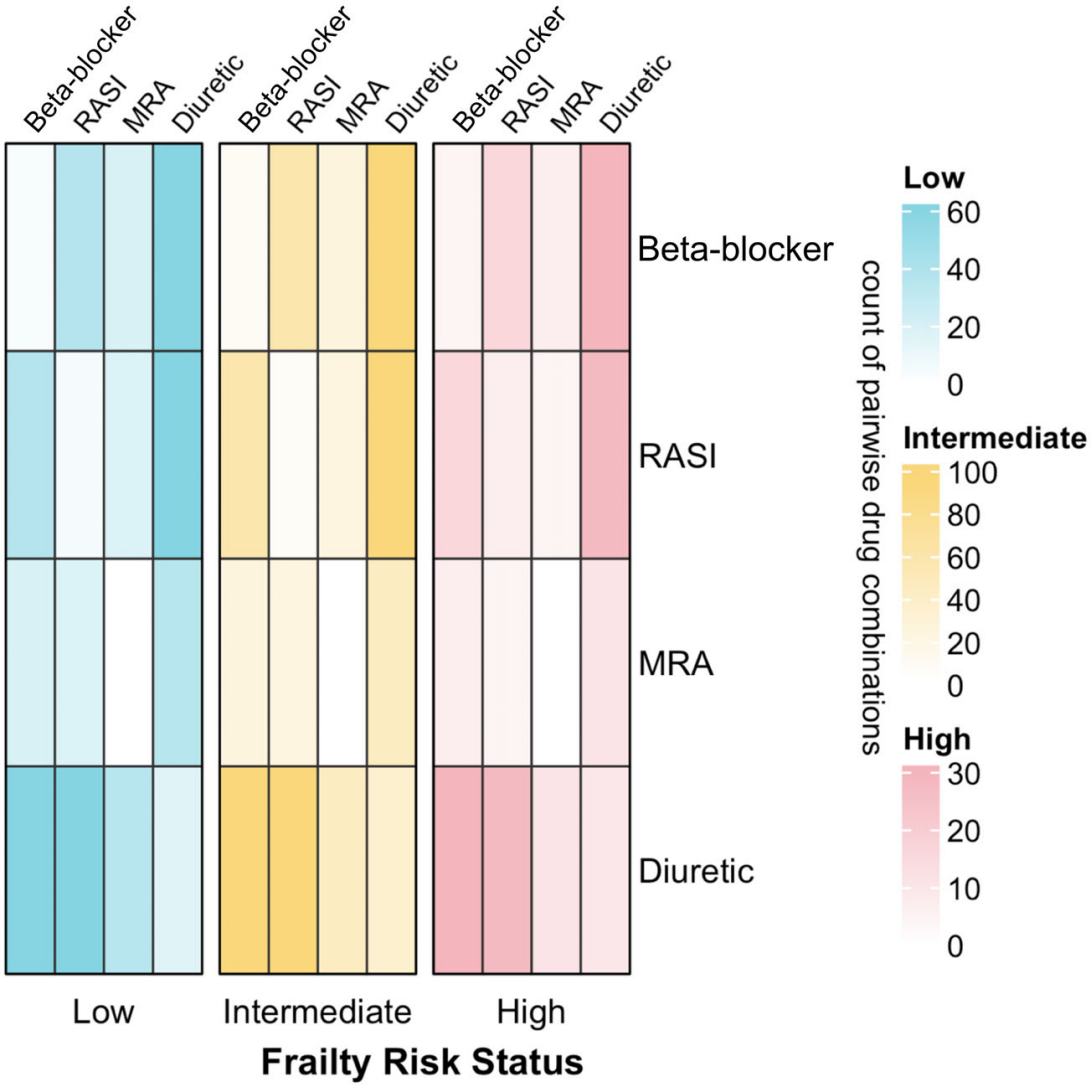
Heatmap displaying frequencies of dual heart failure pharmacotherapy combinations in hospitalised patients aged over 75 years, by frailty risk. Same drug class (diagonals) represents the frequencies of monotherapy and not duplicate therapy. Shaded areas represent the proportion of dual heart failure pharmacotherapy combinations per frailty risk group (low: *n* = 134, tntermediate: *n* = 230, high: *n* = 75); MRA, mineralocorticoid receptor antagonist; RASI, renin‐angiotensin system inhibitor.

Polypharmacy (*n* = 411, 93.6%) and hyperpolypharmacy (*n* = 219, 50.0%) at admission increased at discharge (*n* = 426, 97.0% and *n* = 281, 64.0%, respectively) (Table S2). The proportion of HF patients with hyperpolypharmacy was 10% higher in those with high compared to low frailty risk (high 56.0%, intermediate 50.4%, low 45.5%). Eighty‐eight (20.0%) patients with HF had no HF GDMT, of whom 27 (6.2%) were on neither HF GDMT nor diuretic (Table S2). Monotherapy diuretic use was reported in 61 (13.9%) patients. Multidrug combinations with diuretics were frequent. Use of two (*n* = 170, 41.3%) or three (*n* = 118, 28.6%) HF pharmacotherapies were most frequent, but use of four (*n* = 25, 6.1%) was uncommon (Table S2). The most frequent pairwise HF pharmacotherapy combinations were diuretic–beta‐blocker (*n* = 184, 44.7%), diuretic–RASI (*n* = 183, 44.4%) and beta‐blocker–RASI (*n* = 112, 27.2%) combinations, while monotherapies were uncommon (Fig. 3). This pattern of utilisation of dual HF pharmacotherapy combinations is similar across frailty risk groups (Table S3).

### Clinical outcomes and adverse drug events

**Figure 4:**
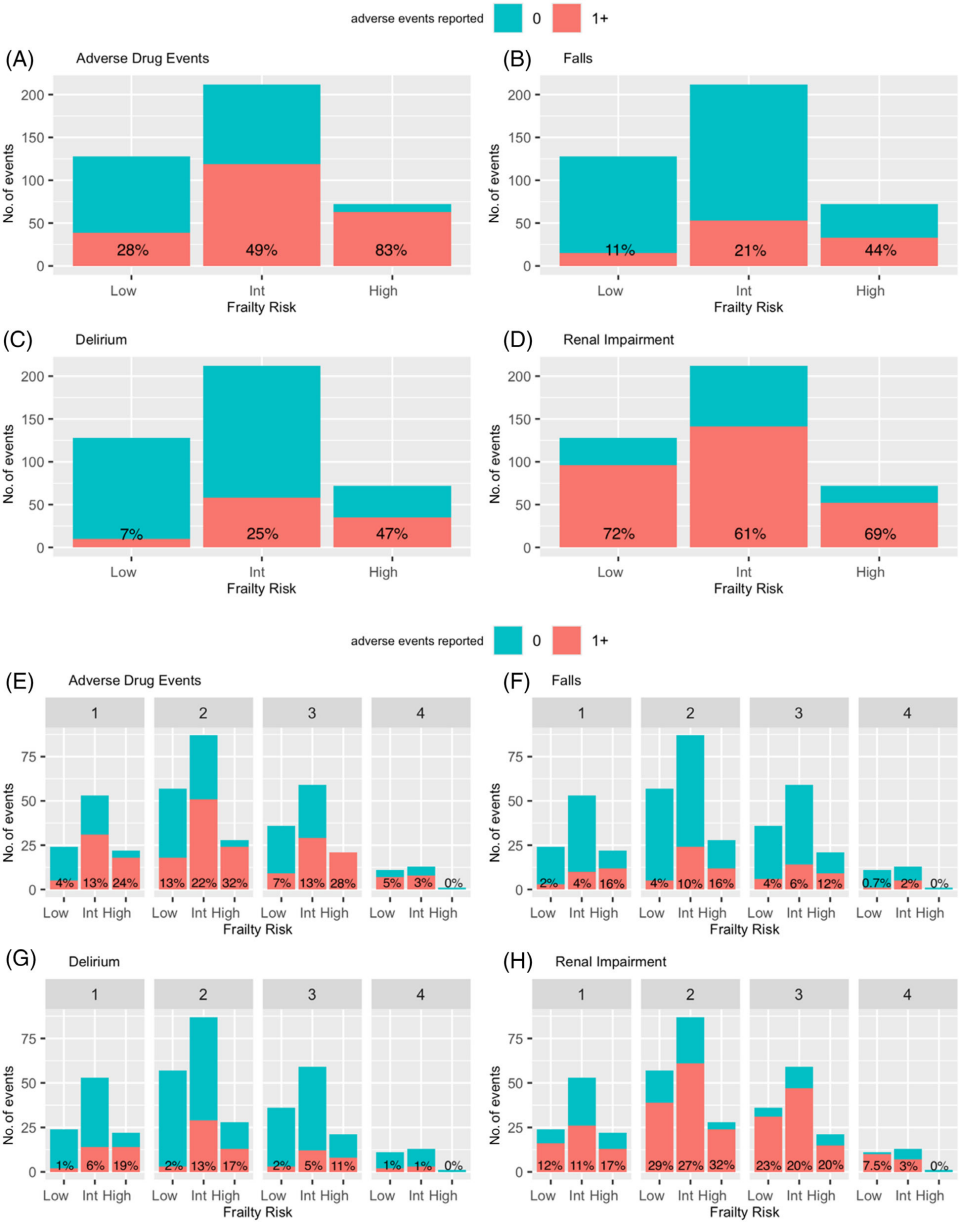
Frequency of adverse drug events and adverse clinical outcomes in hospitalised patients over 75 years on at least one heart failure pharmacotherapy (A–D); and on monotherapy or two, three or four combination heart failure pharmacotherapies (E–H), according to frailty status. Int, intermediate frailty risk. Annotated percentage represents the proportion of adverse events (red) per frailty risk group (low: *n* = 134; intermediate: *n* = 230; high: *n* = 75).

Hospital LOS increased with increasing frailty among HF patients with low (7.8 (6.9) days), intermediate (14.7 (12.9) days) and high (20.3 (15.3) days) frailty risk, and up to 2.6‐fold longer in high‐ compared to low‐frailty‐risk groups. ADEs were high, occurring in approximately half of patients with HF (*n* = 227, 51.7%). Compared to the proportion in patients with low frailty risk (*n* = 40, 29.9%), the prevalence of ADEs was 1.8‐fold higher (*n* = 124, 53.9%) in intermediate‐ and 2.8‐fold higher (*n* = 63, 84.0%) in high‐frailty‐risk patients (Figs 4, S2, Table S5).

Similar prevalence of falls (*n* = 106, 24.1%) and delirium (*n* = 110, 25.1%) were reported in patients with HF; of whom 25 (6.1%) patients experienced both falls and delirium, but mostly occurred in those with high frailty risk (*n* = 17, 68.0%). Compared to the proportion in patients with low frailty risk (*n* = 15, 11.2%), falls were 2.2‐fold higher (*n* = 57, 24.8%) in intermediate and fourfold higher (*n* = 34, 45.3%) in high frailty risk. Compared to the proportion in patients with low frailty risk (*n* = 10, 7.5%), delirium was 3.7‐fold higher (*n* = 63, 27.4%) in intermediate and 6.6‐fold higher (*n* = 37, 49.3%) in high frailty risk. Renal impairment (*n* = 305, 69.5%) in patients with HF was similar across frailty risk groups, ranging between 66% and 74%, and was often prevalent in patients using ≥2 HF‐pharmacotherapies (Fig. 4).

In frequent pairwise‐combinations, the proportions of ADEs in people with high frailty risk increased by 3.2‐fold for diuretic–beta‐blocker (*n* = 29/30, 97%; *n* = 18/60, 30%), 2.3‐fold for diuretic–RASI (*n* = 24/28, 86%; *n* = 23/61, 38%) and 2.5‐fold for beta‐blocker–RASI (*n* = 14/16, 88%; *n* = 13/37, 35%) combinations compared to low frailty risk (Fig. 3, Table S4).

## Discussion

This study demonstrated that among hospitalised patients over 75 years with HF, most were at risk of frailty; diuretics were frequently continued, uptitrated or initiated during hospitalisation; beta‐blockers were mostly continued at discharge; and few were prescribed MRAs. Older patients with HF at high risk of frailty frequently discontinued RASIs compared to those with low frailty risk. Polypharmacy, hyperpolypharmacy and combination use of diuretic–beta‐blocker and diuretic–RASI were frequent; ADEs were highly prevalent among those with high frailty risk. Prevalence of combination therapy was lower in patients with high frailty risk, potentially due to reduced tolerance. HF patients with increasing frailty risk reported longer hospitalisation and increasing proportions of ADEs, falls and delirium. Renal impairment was common across all frailty subgroups. Barriers to evaluating evidence‐based medication management during changes in hospital were identified.

This study provides new insights on the prevalence of ADEs, falls, delirium and renal impairment in a geriatric hospital population with HF and frailty risk. The adverse clinical outcomes reported in this study can significantly impact the quality of life in people of advanced age with frailty, who may no longer prioritise longevity and have more difficulty tolerating HF pharmacotherapy or recovering from ADEs. A systematic review of ADEs associated with HF pharmacotherapy in older people with HF and frailty reported a twofold increased risk of mortality and hospitalisation in frail compared to robust people on RASIs,^15^ but overall there were no data for adverse geriatric outcomes. These findings support consideration of global function and patient preference during medication management of HF in people with advanced age and frailty who may prefer to optimise quality of life and avoid hospitalisation. In addition, these patients may benefit from uptake of strategies to reverse frailty risk.

The ADEs and adverse clinical outcomes reported in this study are important to consider within the context of potential benefits of HF pharmacotherapy. Findings showed increased numbers of pharmacotherapies increased the prevalence of ADEs, falls and delirium with increasing frailty risk. A recent study^32^ conducted in Taiwan (*n* = 38 843) reported a similar frailty distribution and showed 27.3% of HF patients died of cardiovascular diseases. Compared to robust patients with HF, patients with severe frailty had increased risk of all‐cause mortality (adjusted hazard ratio (aHR) 1.16, 95% confidence interval (CI) 1.11–1.21) and all‐cause re‐admissions (subdistributional hazard ratio 1.21, 95% CI 1.16–1.25).^32^ Use of any triple HF pharmacotherapy or more combinations compared to monotherapy was shown to decrease risk of 2‐year all‐cause re‐admissions (adjusted odds ratio (aOR) 0.49, 95% CI 0.44–0.54) across all frailty groups.^32^ In addition, older patients hospitalised with ADRs often have more comorbidities (mean 6.1) compared to those without ADRs (mean 5.2).^11^ In the global REPORT‐HF study (*n* = 18 553), patients presenting with acute HF and a higher number (≥5) of comorbidities (*n* = 4449, 24%) were more likely to use potentially HF‐worsening medications and were less likely to receive HF GDMTs (i.e. RASI, MRA) at discharge.^33^ Thus, the benefit and risks of these medications should be personalised for each individual when supporting optimisation in people with HF, multimorbidity and high frailty risk.

Barriers to evidence‐based management of changes during hospitalisation included poor documentation of HF phenotype and rationale for deprescribing. Appropriate medication optimisation could not be evaluated in the absence of accurate HF diagnosis. The frequency of deprescribing (~40%) was similar among patients with acute and chronic stable HF but did not distinguish whether deprescribing was intended to be permanent or if medications were temporarily ceased to manage acute issues. Specific reasons for deprescribing were not captured in this dataset; however, potential reasons include management of hypotension, acute kidney injury, syncope, falls, drug interactions, infections and changes in cognition, for example. Among community aged care residents with dementia, deprescribing of anti‐hypertensives (without HF diagnosis) was associated with 16% reduced odds of cognitive decline (OR 0.84, 95% CI 0.72–0.98) compared with continued use in usual care.^34^ Future investigation is needed to explore safety and rationale for deprescribing HF pharmacotherapy or potentially high‐risk medications^39^, ^35^ in this population.

### Strengths and limitations

This descriptive study provides novel data on adverse geriatric outcomes in older hospitalised adults with HF and frailty risk that can be applied to the optimisation and management of adverse events by multidisciplinary teams. In a cohort of older people, adverse outcomes were particularly high in patients with high frailty risk, supporting the need to consider not just age but global function in shared decision‐making. The proportion of 22% with HF diagnosis in the TO HOME sample is comparable to other geriatric populations internationally.^36^, ^37^, ^38^ The methodology used in this study can be applied to future investigations of other diseases (e.g. atrial fibrillation) or diverse populations to report geriatric outcomes by frailty risk.

Data were not available to determine causal relationships between medication and adverse reactions, and there was no confirmation on whether adverse outcomes were caused by HF pharmacotherapy, non‐specific or related to other potentially inappropriate medications treating comorbidities. The association or causality of underutilisation or optimised HF pharmacotherapy utilisation with adverse outcomes, mortality and re‐admission was not investigated. The frequency of ADEs in pairwise combinations was counted per patient, and retrospective ICD‐10‐AM diagnosis codes could contribute to both frailty risk and the associated outcomes, specifically for delirium and falls. Evaluation of SGLT2‐i and emerging therapies (e.g. vericiguat, omecamtiv mecarbil) and subanalysis of potential confounders (e.g. age, sex, renal function, ethnicity) were not performed due to small sample size. Patients admitted under cardiology service were not included in the TO HOME study. Cardiovascular biomarkers and HF phenotypes were not included at the time of data collection. The small sample size in an urban Australian inpatient population with 25% from residential aged care settings limited the generalisability of the results. Future prospective cohort studies and clinical trials of HF pharmacotherapy utilisation and optimisation in older people should build on our findings by considering the inclusion of these variables and analyses in a larger more diverse population.

## Conclusions

This study demonstrated that older adults with HF and frailty risk were frequently prescribed diuretics, diuretic–GDMT combinations and continued beta‐blockers during hospitalisation. HF phenotype was not documented, and it is unknown whether it guided therapeutic choices. The reasons for the lack of documentation in this older population should be explored. A higher proportion of people with high frailty risk discontinued RASIs. Polypharmacy and renal impairment were prevalent across all frailty groups. Duration of hospitalisation and reported prevalence of ADEs, falls and delirium increased with increased frailty risk. These findings can be considered when managing cardiovascular outcomes in complex older patients with limited global function. Research reporting adverse outcomes of mortality, hospitalisation, cardiovascular biomarkers, geriatric outcomes and frailty is needed to inform shared decision‐making and advanced care planning.

## Supporting information


**Data S1** Supporting Information.
