# Supplementary material for: Prevalence of heart failure pharmacotherapy utilisation, frailty and adverse drug events among hospitalised adults older than 75 years: a multicentre cross‐sectional study
Source: Intern Med J. 2024 Dec 19;55(2):249–59. doi: 10.1111/imj.16612 (PMC11817903; doi:10.1111/imj.16612)
Supplement: Supplementary file 1 — Data S1 Supporting Information. [file IMJ-55-249-s001.pdf]

**Supplementary Online Materials**

**Figure S1** The proportion of hospitalised patients aged over 75 years with heart failure pharmacotherapy status change**,** stratified by their risk of frailty.

**Table S1** The frequency of heart failure pharmacotherapy utilisation that was initiated, continued, up-titrated, down-titrated, discontinued, and re-initiated at discharge by inpatients over 75 years with heart failure according to frailty status.

**Table S2** Polypharmacy and combination heart failure pharmacotherapy use at discharge in older inpatients according to frailty risk.

**Table S3** Frequency of pairwise drug class combination use according to frailty status.

**Table S4** Frequency of adverse drug events (n) per pairwise drug class combination (N) according to frailty status.

**Figure S2** Frequency and proportion of heart failure-pharmacotherapy pairwise-combinations and adverse drug events in hospitalised patients aged over 75 years**,** by frailty risk status.

**Table S5** The frequency of adverse outcomes in adults $\geq$75 years with HF at risk of frailty on GDMT and diuretics

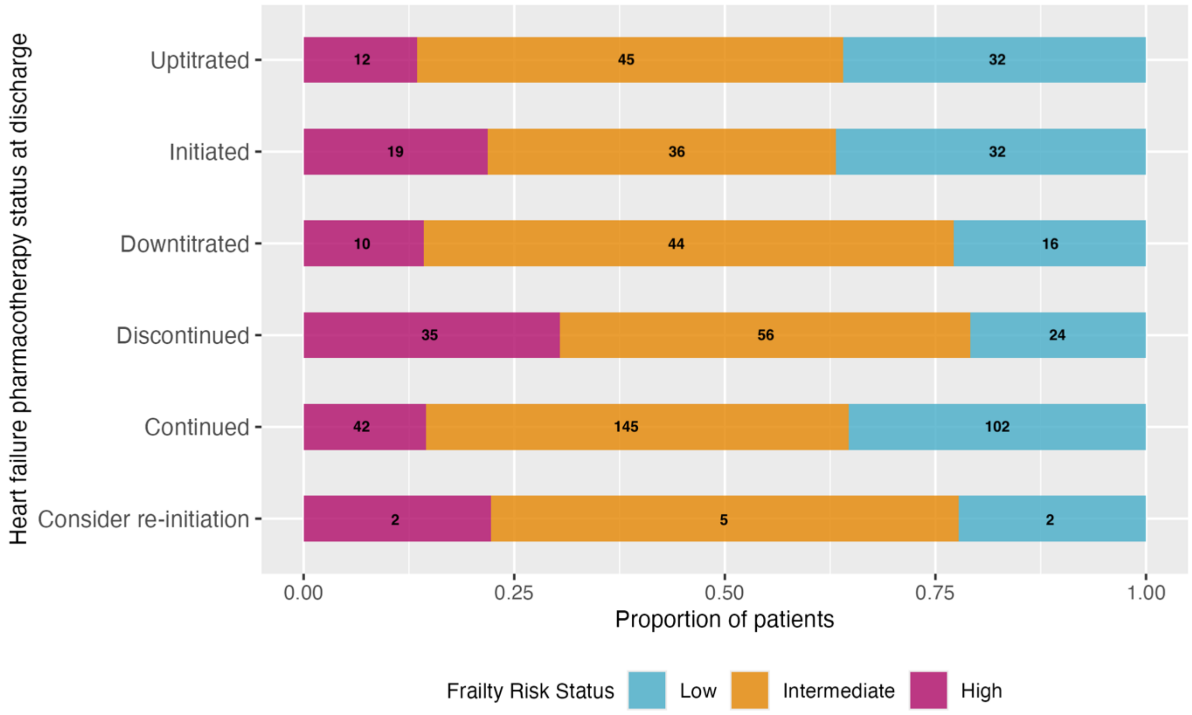


**Figure S1.** The proportion of hospitalised patients over 75 years with heart failure pharmacotherapy status change, stratified by their risk of frailty.

*Footnote: annotated numbers represent the number of patients.*

| | Low Risk Frailty (HFRS<5) | | Moderate Risk Frailty (HFRS 5-15) | | High Risk Frailty (HFRS>15) | |
|---|---|---|---|---|---|---|
| | *n* | % | *n* | % | *n* | % |
| **Initiated** | | | | | | |
| **at least 1 GDMT or diuretic** | 34 | 8% | 37 | 8% | 19 | 4% |
| ACEI/ARB | 9 | 2.1% | 6 | 1.4% | 3 | 0.7% |
| Beta blocker | 3 | 0.7% | 2 | 0.5% | 3 | 0.7% |
| MRA | 1 | 0.2% | 1 | 0.2% | 2 | 0.5% |
| Loop diuretic | 26 | 5.9% | 28 | 6.4% | 14 | 3.2% |
| **Continued** | | | | | | |
| **at least 1 GDMT or diuretic** | 102 | 23% | 145 | 33% | 42 | 10% |
| ACEI/ARB | 41 | 9.3% | 53 | 12.1% | 12 | 2.7% |
| Beta blocker | 53 | 12.1% | 81 | 18.5% | 26 | 5.9% |
| MRA | 23 | 5.2% | 34 | 7.7% | 5 | 1.1% |
| Loop diuretic | 56 | 12.8% | 71 | 16.2% | 15 | 3.4% |
| **Up-titrated** | | | | | | |
| **at least 1 GDMT or diuretic** | 32 | 7.3% | 45 | 10.3% | 12 | 2.7% |
| ACEI/ARB | 2 | 0.5% | 4 | 0.9% | 0 | 0.0% |
| Beta blocker | 2 | 0.5% | 5 | 1.1% | 2 | 0.5% |
| MRA | 7 | 1.6% | 2 | 0.5% | 0 | 0.0% |
| Loop diuretic | 24 | 5.5% | 40 | 9.1% | 10 | 2.3% |
| **Down-titrated** | | | | | | |
| **at least 1 GDMT or diuretic** | 16 | 3.6% | 44 | 10.0% | 10 | 2.3% |
| ACEI/ARB | 3 | 0.7% | 7 | 1.6% | 1 | 0.2% |
| Beta blocker | 5 | 1.1% | 14 | 3.2% | 3 | 0.7% |
| MRA | 2 | 0.5% | 1 | 0.2% | 1 | 0.2% |
| Loop diuretic | 8 | 1.8% | 28 | 6.4% | 5 | 1.1% |
| **Discontinued** | | | | | | |
| **at least 1 GDMT or diuretic** | 24 | 5.5% | 56 | 12.8% | 35 | 8.0% |
| ACEI/ARB | 17 | 3.9% | 37 | 8.4% | 21 | 4.8% |
| Beta blocker | 5 | 1.1% | 7 | 1.6% | 3 | 0.7% |
| MRA | 3 | 0.7% | 10 | 2.3% | 5 | 1.1% |
| Loop diuretic | 2 | 0.5% | 16 | 3.6% | 12 | 2.7% |
| **Re-initiated at discharge** | 0, 0% for all therapeutic classes | | | | | |
| **Withheld on admission, GP review before restarting** | | | | | | |
| **at least 1 GDMT or diuretic** | 2 | 0.5% | 5 | 1% | 2 | 0.5% |
| ACEI/ARB | 1 | 0.5% | 2 | 0.7% | 0 | 0.2% |
| Beta blocker | 0 | 0.0% | 1 | 0.2% | 1 | 0.2% |
| MRA | 0 | 0.0% | 2 | 0.7% | 0 | 0.0% |
| Loop diuretic | 1 | 0.2% | 0 | 0.7% | 1 | 0.2% |

**Table S1:** The frequency of heart failure pharmacotherapy utilisation that was initiated, continued, up-titrated, down-titrated, discontinued, and re-initiated at discharge by inpatients over 75 years with heart failure according to frailty status.

*Abbreviations: GP General Practitioner; GDMT Guideline-Directed Medical Therapy; ACEI angiotensin converting enzyme inhibitor; ARB angiotensin receptor antagonist; MRA Mineralocorticoid Antagonist*
*Footnote: Initiation includes status coded as "Other" and "New medication".*

| | Total | | Low Risk Frailty (HFRS <5) | | Moderate Risk Frailty (HFRS 5-15) | | High Risk Frailty (HFRS >15) | |
|---|---|---|---|---|---|---|---|---|
| | *n* | % | *n* | % | *n* | % | *n* | % |
| **Sample size** | **439** | **100** | **134** | **30.5%** | **230** | **52.4%** | **75** | **17.1%** |
| **Medication Class** | | | | | | | | |
| Loop diuretic | 357 | 81.3% | 115 | 85.8% | 185 | 80.4% | 57 | 76.0% |
| **Guideline-Directed Medical Therapy (GDMT)*** | | | | | | | | |
| ACEI/ARB | 218 | 49.7% | 71 | 53.0% | 110 | 47.8% | 37 | 49.3% |
| Beta blocker | 216 | 49.2% | 68 | 50.7% | 110 | 47.8% | 38 | 50.7% |
| Mineralocorticoid Antagonist | 100 | 22.8% | 36 | 26.9% | 51 | 22.2% | 13 | 17.3% |
| **Combinations use of GDMT** | | | | | | | | |
| 0 | 88 | 20.0% | 22 | 5.0% | 53 | 12.1% | 13 | 3.0% |
| 1 | 196 | 44.6% | 61 | 13.9% | 97 | 22.1% | 38 | 8.7% |
| 2 | 127 | 28.9% | 39 | 8.9% | 66 | 15.0% | 22 | 5.0% |
| 3 | 28 | 6.4% | 12 | 2.7% | 14 | 3.2% | 2 | 0.5% |
| **Combinations use heart failure pharmacotherapy (GDMT or diuretic)** | | | | | | | | |
| 0 | 27 | 6.6% | 6 | 4.5% | 18 | 7.8% | 3 | 4.0% |
| 1 | 99 | 24.0% | 24 | 17.9% | 53 | 23.0% | 22 | 29.3% |
| 2 | 170 | 41.3% | 56 | 41.8% | 86 | 37.4% | 28 | 37.3% |
| 3 | 118 | 28.6% | 37 | 27.6% | 60 | 26.1% | 21 | 28.0% |
| 4 | 25 | 6.1% | 11 | 8.2% | 13 | 5.7% | 1 | 1.3% |
| **Number of medications at admission** | | | | | | | | |
| No Polypharmacy (<5 medications) | 28 | 6.4% | 8 | 6.0% | 14 | 6.1% | 6 | 8.0% |
| Polypharmacy (≥5 medications) | 411 | 93.6% | 126 | 94.0% | 216 | 93.9% | 69 | 92.0% |
| Hyperpolypharmacy (≥10 medications) | 219 | 49.9% | 61 | 45.5% | 116 | 50.4% | 42 | 56.0% |
| **Number of medications at discharge** | | | | | | | | |
| No Polypharmacy (<5 medications) | 13 | 3.0% | 1 | 0.7% | 10 | 4.3% | 2 | 2.7% |
| Polypharmacy (≥5 medications) | 426 | 97.0% | 133 | 99.3% | 220 | 95.7% | 73 | 97.3% |
| Hyperpolypharmacy (≥10 medications) | 281 | 64% | 74 | 55.2% | 154 | 67.0% | 53 | 70.7% |

**Table S2:** Polypharmacy and combination heart failure pharmacotherapy use at discharge in older inpatients according to frailty risk.

*Abbreviation: HFRS Hospital Frailty Risk Score; ACEI angiotensin converting enzyme inhibitor; ARB angiotensin receptor antagonist;*
*Footnote: Diuretics predominantly refer to furosemide (n=355), etacrynic acid (n=1) and butanamide (n=1).*

| | | Beta blocker | | RASI | | MRA | | Loop diuretic | |
|---|---|---|---|---|---|---|---|---|---|
| | | **(*n*)** | **(%)** | **(*n*)** | **(%)** | **(*n*)** | **(%)** | **(*n*)** | **(%)** |
| ***Low Frailty*** *(n=134)* | **Beta blocker** | 3 | 2.2% | 37 | 27.6% | 20 | 14.9% | 60 | 44.8% |
| | **RASI** | 37 | 27.6% | 5 | 3.7% | 18 | 13.4% | 61 | 45.5% |
| | **MRA** | 20 | 14.9% | 18 | 13.4% | 0 | 0.0% | 35 | 26.1% |
| | **Loop diuretic** | 60 | 44.8% | 61 | 45.5% | 35 | 26.1% | 16 | 11.9% |
| ***Intermediate Frailty*** *(n=230)* | **Beta blocker** | 9 | 3.9% | 59 | 25.7% | 25 | 10.9% | 94 | 40.9% |
| | **RASI** | 59 | 25.7% | 7 | 3.0% | 24 | 10.4% | 94 | 40.9% |
| | **MRA** | 25 | 10.9% | 24 | 10.4% | 2 | 0.9% | 46 | 20.0% |
| | **Loop diuretic** | 94 | 40.9% | 94 | 40.9% | 46 | 20.0% | 35 | 15.2% |
| ***High Frailty*** *(n=75)* | **Beta blocker** | 5 | 6.7% | 16 | 21.3% | 7 | 9.3% | 30 | 40.0% |
| | **RASI** | 16 | 21.3% | 7 | 9.3% | 5 | 6.7% | 28 | 37.3% |
| | **MRA** | 7 | 9.3% | 5 | 6.7% | 0 | 0.0% | 11 | 14.7% |
| | **Loop diuretic** | 30 | 40.0% | 28 | 37.3% | 11 | 14.7% | 10 | 13.3% |

**Table S3:** Frequency of pairwise drug class combination use according to frailty status

*Abbreviation: RASI Renin Angiotensin System Inhibitor; MRA Mineralocorticoid Receptor Antagonist*
*Footnote: Values are reported per drug-class (not per patient), which may be repeated for patients on three of four HF pharmacotherapies*

| | | Beta blocker | | | RASI | | | MRA | | | Loop diuretic | | |
|---|---|---|---|---|---|---|---|---|---|---|---|---|---|
| | | (*n*) | (N) | (%) | (*n*) | (N) | (%) | (*n*) | (N) | (%) | (*n*) | (N) | (%) |
| ***Low Frailty*** *(n=134)* | **Beta blocker** | 0 | 3 | 0% | 13 | 37 | 35% | 9 | 20 | 45% | 18 | 60 | 30% |
| | **RASI** | 13 | 37 | 35% | 1 | 5 | 20% | 9 | 18 | 50% | 23 | 61 | 38% |
| | **MRA** | 9 | 20 | 45% | 9 | 18 | 50% | 0 | 0 | 0% | 15 | 35 | 43% |
| | **Loop diuretic** | 18 | 60 | 30% | 23 | 61 | 38% | 15 | 35 | 43% | 0 | 16 | 0% |
| ***Intermediate Frailty*** *(n=230)* | **Beta blocker** | 5 | 9 | 56% | 29 | 59 | 49% | 13 | 25 | 52% | 51 | 94 | 54% |
| | **RASI** | 29 | 59 | 52% | 1 | 7 | 14% | 14 | 24 | 58% | 53 | 94 | 56% |
| | **MRA** | 13 | 25 | 52% | 14 | 24 | 58% | 1 | 2 | 50% | 26 | 46 | 57% |
| | **Loop diuretic** | 51 | 94 | 54% | 53 | 94 | 56% | 26 | 46 | 57% | 0 | 35 | 0% |
| ***High Frailty*** *(n=75)* | **Beta blocker** | 5 | 5 | 100% | 14 | 16 | 88% | 6 | 7 | 86% | 29 | 30 | 97% |
| | **RASI** | 14 | 16 | 88% | 0 | 7 | 0% | 4 | 5 | 80% | 24 | 28 | 86% |
| | **MRA** | 6 | 7 | 86% | 4 | 5 | 80% | 0 | 0 | 0% | 10 | 11 | 91% |
| | **Loop diuretic** | 29 | 30 | 97% | 24 | 28 | 86% | 10 | 11 | 91% | 0 | 10 | 0% |

**Table S4:** Frequency of adverse drug events (*n*) per pairwise drug class combination (N) according to frailty status

*Abbreviations: RASI Renin Angiotensin System Inhibitor; MRA Mineralocorticoid Receptor Antagonist;*
*Footnote: Values are reported per drug-class (not per patient), which may be repeated for patients on three of four HF pharmacotherapies; n count of adverse drug events reported; N count of pairwise drug class combinations; % the proportion of adverse drug events per pairwise drug class combinations (n/N*100%)*

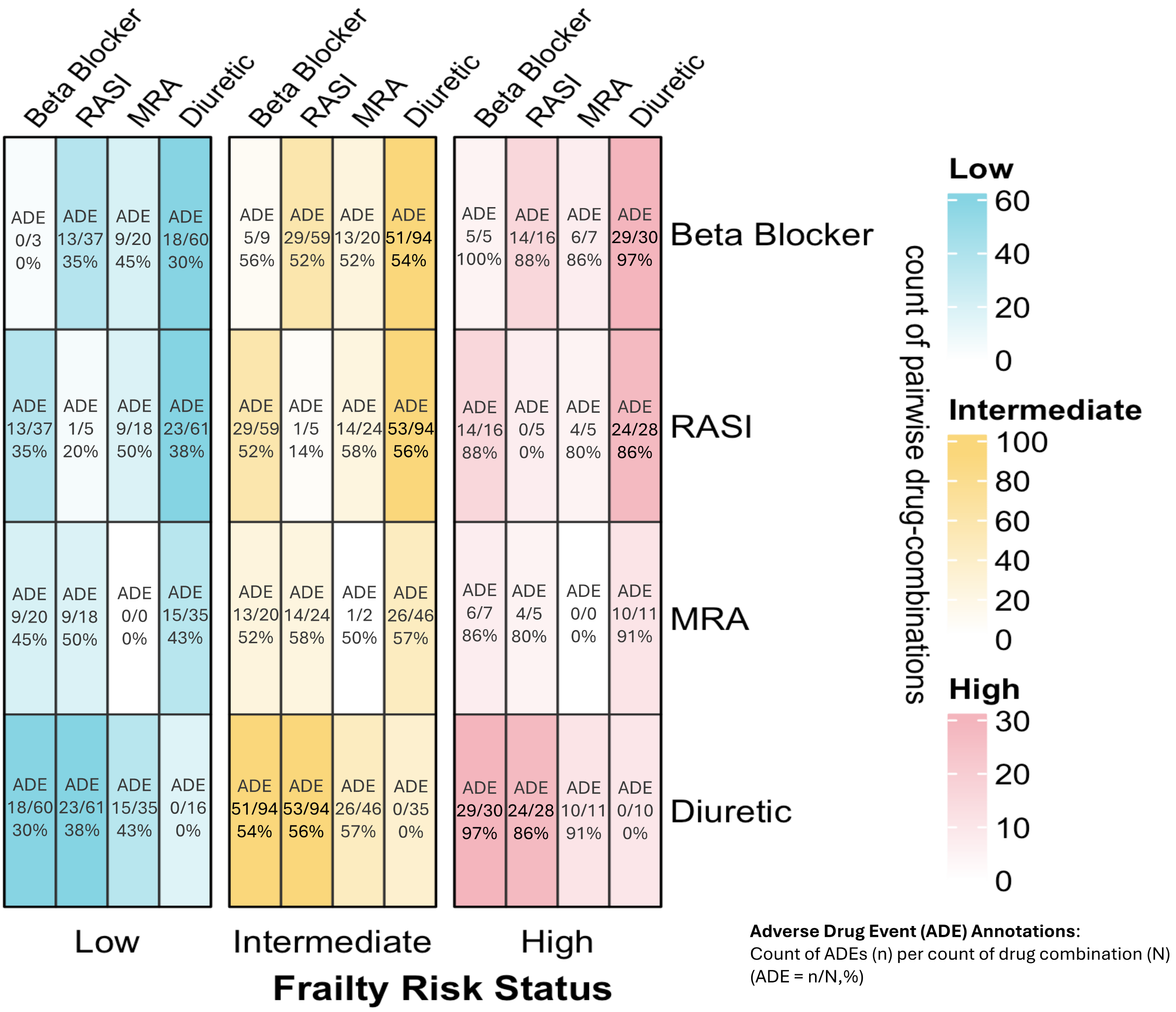


**Figure S1.** Frequency and proportion of adverse drug events in hospitalised patients aged over 75 years with heart failure-pharmacotherapy pairwise-combinations**,** by frailty risk status.

*Footnote: Same drug class (Diagonals) represent the frequencies of monotherapy and not duplicate therapy. Shaded areas represent the proportion of dual heart failure-pharmacotherapy combinations per frailty risk group (Low: n=134, Int: n=230, High: n=75); Annotated percentage represents the proportion of adverse events per frailty risk group (Low: n=134, Int: n=230, High: n=75);*
*Abbreviations: HF Heart failure; ADE Adverse Drug Event; HF Heart Failure, RASI Renin Angiotensin System Inhibitors; MRA Mineralocorticoid Receptor Antagonists.*

| | Total HF population | | | ≥1 GDMT or diuretic | | | HF-Drug Class | | | |
|---|---|---|---|---|---|---|---|---|---|---|
| | **(*n*)** | **(%)** | **%HF Frailty group** | **(*n*)** | **(%)** | **%HF Frailty group** | **Beta blocker** | **RASI** | **MRA** | **Loop Diuretic** |
| | **439** | **22%** | | | | | **216** | **218** | **100** | **357** |
| **Low Frailty Risk (HFRS <5)** | **134** | **31%** | **100%** | **128** | 29% | 96% | | | | |
| Number of ADEs | 40 | 9% | 30% | 39 | 9% | 28% | 68 | 71 | 36 | 115 |
| Falls | 15 | 3% | 11% | 15 | 3% | 11% | 8 | 11 | 2 | 14 |
| Delirium | 10 | 2% | 7% | 10 | 2% | 7% | 6 | 6 | 4 | 9 |
| Renal Impairment (GFR<60mL/min/1.72m$^2$) | 99 | 23% | 74% | 96 | 22% | 72% | 55 | 50 | 32 | 79 |
| **Moderate Frailty Risk (HFRS 5-15)** | **230** | **52%** | **100%** | **212** | 48% | 92% | | | | |
| Number of ADEs | 124 | 28% | 54% | 119 | 26% | 49% | 110 | 110 | 51 | 185 |
| Falls | 57 | 13% | 25% | 48 | 11% | 21% | 28 | 30 | 16 | 46 |
| Delirium | 63 | 14% | 27% | 58 | 13% | 25% | 28 | 25 | 13 | 54 |
| Renal Impairment* (GFR<60mL/min/1.72m$^2$) | 151 | 34% | 66% | 141 | 32% | 61% | 79 | 75 | 35 | 128 |
| **High Frailty Risk (HFRS >15)** | **75** | **17%** | **100%** | **72** | 16% | 96% | | | | |
| Number of ADEs | 63 | 14% | 84% | 63 | 14% | 83% | 38 | 37 | 13 | 57 |
| Falls | 34 | 8% | 45% | 33 | 8% | 44% | 17 | 16 | 4 | 26 |
| Delirium | 37 | 8% | 49% | 35 | 8% | 47% | 15 | 18 | 3 | 28 |
| Renal Impairment (GFR<60mL/min/1.72m$^2$) | 55 | 13% | 73% | 52 | 12% | 69% | 26 | 29 | 9 | 42 |

**Table S5:** The frequency of adverse outcomes in adults ≥75 years with HF at risk of frailty on GDMT and diuretics.
*Abbreviation: HF Heart Failure, GDMT Guideline Directed Medical Therapy; HFRS Hospital Frailty Risk Score; ADE Adverse Drug Event; RASI Renin Angiotensin System Inhibitors; MRA Mineralocorticoid Receptor Antagonists; GFR Glomerular Filtration Rate. Footnote: Falls, delirium, renal impairment were measured according to ICD-10 and considered separate diagnosis from ADEs. *1 person in moderate frailty group was coded as 9999 = missing data coded as no renal impairment.*
